# Probiotic *Lactobacillus fermentum* TSF331, *Lactobacillus reuteri* TSR332, and *Lactobacillus plantarum* TSP05 improved liver function and uric acid management-A pilot study

**DOI:** 10.1371/journal.pone.0307181

**Published:** 2024-07-24

**Authors:** Jia-Hung Lin, Chi-Huei Lin, Yi-Wei Kuo, Chorng-An Liao, Jui-Fen Chen, Shin-Yu Tsai, Ching-Min Li, Yu-Chieh Hsu, Yen-Yu Huang, Ko-Chiang Hsia, Yao-Tsung Yeh, Hsieh-Hsun Ho

**Affiliations:** 1 Functional R&D Department, Research and Design Center, Glac Biotech Co., Ltd., Tainan City, Taiwan; 2 Aging and Disease Prevention Research Center, Fooyin University, Kaohsiung City, Taiwan; 3 Research Product Department, Research and Design Center, Glac Biotech Co., Ltd., Tainan City, Taiwan; 4 Department of Medical Laboratory Sciences and Biotechnology, Fooyin University, Kaohsiung City, Taiwan; Fonterra Coop / Lebanese University, LEBANON

## Abstract

Metabolic-associated fatty liver disease (MAFLD) is predominantly associated with metabolic disturbances representing aberrant liver function and increased uric acid (UA) levels. Growing evidences have suggested a close relationship between metabolic disturbances and the gut microbiota. A placebo-controlled, double-blinded, randomized clinical trial was therefore conducted to explore the impacts of daily supplements with various combinations of the probiotics, *Lactobacillus fermentum* TSF331, *Lactobacillus reuteri* TSR332, and *Lactobacillus plantarum* TSP05 with a focus on liver function and serum UA levels. Test subjects with abnormal levels of aspartate aminotransferase (AST), alanine aminotransferase (ALT), and UA were recruited and randomly allocated into six groups. Eighty-two participants successfully completed the 60-day intervention without any dropouts or occurrence of adverse events. The serum AST, ALT, and UA levels were significantly reduced in all treatment groups (*P* < 0.05). The fecal microbiota analysis revealed the intervention led to an increase in the population of commensal bacteria and a decrease in pathobiont bacteria, especially *Bilophila wadsworthia*. The *in vitro* study indicated the probiotic treatments reduced lipid accumulation and inflammatory factor expressions in HepG2 cells, and also promoted UA excretion in Caco-2 cells. The supplementation of multi-strain probiotics (TSF331, TSR332, and TSP05) together can improve liver function and UA management and may have good potential in treating asymptomatic MAFLD.

**Trial registration**. The trial was registered in the US Library of Medicine (clinicaltrials.gov) with the number NCT06183801 on December 28, 2023.

## Introduction

Urbanization leads to higher rates of metabolic disturbances due to changes in diet, reduced physical activity, and increased stress. Metabolic disturbances commonly result in obesity, insulin resistance, and dyslipidemia. Fatty liver disease (FLD) is a condition where excess fat builds up in the liver, and there are two types of FLDs: non-alcoholic fatty liver disease (NAFLD) and alcoholic liver disease (ALD) [1]. FLD is a spectrum, with the presence of fat accumulation being the mildest form and steatohepatitis and cirrhosis being the worst form [2]. While ALD and NAFLD share similar pathological spectra, their etiological factors diverge significantly. ALD is primarily driven by excessive alcohol intake, whereas NAFLD is associated with overconsumption of food [3]. There is growing evidence that NAFLD is a multisystem disease, affecting extra-hepatic organs and regulatory pathways [4]. Recently, the new term ’metabolic-associated fatty liver disease’ (MAFLD) was proposed to better reflect the metabolism-related etiology [5]. NAFLD, or MAFLD, are asymptomatic, so the combination of serum ALT and AST levels, age, body mass index (BMI), and sex is an important biomarker in the diagnosis of hepatic steatosis [6].

In addition to the three high-risk factors, abnormal UA levels were found to be involved in implications for various medical conditions and became the fourth factor for metabolic syndrome [7]. The relationship between high UA levels and gouty arthritis was well-known for centuries. Recently, high UA levels have been observed to affect inflammation, oxidative stress, as well as enzymes related to lipid and glucose metabolism [8]. Both hyperuricemia and NAFLD are associated with lipid metabolism, and their correlation has been observed in both animal and human studies [9, 10]. Moreover, the association was identified in individuals both with and without obesity. Thus, serum UA levels can be an early indicator for metabolic dysregulation [11, 12]. In other words, effectively managing UA levels is as crucial to human health as managing blood pressure, blood sugar, and blood lipids [13].

Although conflicting results existed in different human clinical studies due to the difficulty to identify all intestinal microbes, the evidence still implicated a link between the gut microbiome and metabolic events [14, 15]. The animal studies demonstrated that FLD development was determined by gut bacteria, and dysbiosis of the gut microbiota was observed in NAFLD patients [16]. Studies have observed different changes in specific taxa correlated with obesity, hyperglycemia, dyslipidemia, hypertension, hyperuricemia, and NAFLD [17]. The development of high-throughput sequencing technologies further illustrates microbial composition differences in diseases [18]. Recent research reveals that dietary lipids favor the growth of *Bilophila*, especially the pathobiont *B*. *wadsworthia*, which can synergize with high fat diet and lead to higher glucose dysmetabolism and hepatic steatosis [19, 20]. The supplementation of prebiotic and probiotic functional foods has drawn much attention as an alternative microbial medicine to maintain healthy metabolism [21]. The health conditions of patients can be improved by probiotic supplementation via reducing serum UA level, fatty liver index, and body weight in clinical trials [22–24]. However, no significant impact of probiotics was reported on the modulation of *Bilophila* or *B*. *wadsworthia*. The positive effects of symbiotics appear to be strain-specific, meaning that a particular symbiotic strain may enhance intestinal health by reshaping the gut microbiota composition without necessarily exhibiting any beneficial effects on liver fat [25, 26]. Therefore, clinical studies are imperative to validate the influence of probiotic functional foods on maintaining human health and microbiota balance, specifically by reducing potential pathobionts.

Prevention is better than cure. A probiotic was defined as “live microorganisms that, when administered in adequate amounts, confer a health benefit on the host” [27]. In recent years, probiotic supplements have been widely used to prevent diseases. *Lactobacillus fermentum* TSF331 and *Lactobacillus reuteri* TSR332 were isolated from healthy human gut. *Lactobacillus plantarum* TSP05 was isolated from Taiwanese pickled cabbage. A study demonstrated *L*. *fermentum* TSF331, *L*. *reuteri* TSR332, and *L*. *plantarum* TSP05 had high antioxidant capacity *in vitro*, and reduced oxidative stress and inflammatory responses in ethanol-induced liver damaged mice [28]. Another study presented *L*. *fermentum* TSF331 and *L*. *reuteri* TSR332 assimilated purine nucleoside without UA generation *in vitro*, and stabilized serum UA levels in oxonate-induced hyperuricemia rats [29]. Therefore, these three *Lactobacillus* strains displayed good potential for liver health and UA management. In addition to probiotics, growing studies demonstrated the postbiotics (probiotic metabolites) played an important role in modulating intestinal microbiota [30, 31].

The aim of this study was to understand whether the supplementation of mono-strain probiotic, multi-strain probiotics, and multi-strain probiotics plus postbiotics displayed different improvements on liver function and UA levels in subjects with a potential risk of MAFLD. Blood samples were collected to monitor the biochemistry profiles, such as AST, ALT and UA levels. Fecal samples were collected to investigate the gut microbiota modulation using NGS analysis. Lipid accumulation and gene expressions of inflammatory factors were examined to elucidate the liver-protective mechanisms in HepG2 cells. UA transporter activity was analyzed to assess UA excretion in Caco-2 cells.

## Materials and methods

### Clinical study

#### Study population

This was a placebo-controlled, double-blinded, randomized clinical study. One hundred and twenty participants were recruited according to their latest physical examination report. The inclusion criteria were ≥ 18 years old, AST > 38 U/L, ALT > 44 U/L, UA > 7 mg/dL for male, and > 6 mg/dL for female. Besides, participants should not have any history of severe liver, cardiovascular, respiratory, kidney disorders or malignancies. Participants were excluded under the following conditions: Firstly, if their serum AST, ALT, or UA levels did not meet the inclusion criteria on day 0. Secondly, if their informed consents were not correctly signed. Thirdly, if their blood test results on day 0 indicated underlying health conditions, such as infections or anemia.

#### Ethical approval

Informed consent was obtained in written form from every subject randomized in the study. The study protocol was approved by the Ethics Committee of Aging and Disease Prevention Research Center at Fooyin University Hospital (FYH-IRB-110-01-02). The recruitment period for this study was from June 1 to December 15, 2021.

#### Study design and sample collection

The placebo capsule contained 500 mg maltodextrin. The mono-strain probiotic capsule contained 6.7 × 10^9^ cfu of either *L*. *fermentum* TSF331, *L*. *reuteri* TSR332, or *L*. *plantarum* TSP05. The 3 Mix capsule contained a total 6.7 × 10^9^ cfu, combining *L*. *fermentum* TSF331, *L*. *reuteri* TSR332, and *L*. *plantarum* TSP05. The 3 Mix+PE0401 capsule was composed of a total 6.7 × 10^9^ cfu, including *L*. *fermentum* TSF331, *L*. *reuteri* TSR332, *L*. *plantarum* TSP05, and 200 mg Totipro^®^ PE0401 postbiotic powder. *L*. *fermentum* TSF331 (BCRC 910815 = CGMCC 15527) and *L*. *reuteri* TSR332 (BCRC 910816 = CGMCC 15528) were isolated from the gut of healthy humans, whereas *L*. *plantarum* TSP05 (BCRC 910855 = CGMCC 16710) was isolated from kimchi. Totipro^®^ PE0401 was a fermentation product derived from probiotics [32]. Capsules were prepared with the same appearance by Glac Biotech Co., Ltd., Taiwan. Every subject was not aware of his/her treatment group, and took 3 capsules per day for 60 days. The blood and fecal samples were collected on day 0 and 60.

#### Blood biochemistry

The blood samples were analyzed by Everest Inspection International Co., Ltd. Seventeen tests were performed to examine the effect of the intervention on physical indexes. The panel of tests measuring liver function included AST, ALT, albumin (ALB), total bilirubin (TBIL), gamma-glutamyl transferase (GGT), and alkaline phosphatase (ALKP). The panel of tests measuring kidney function included UA, creatinine (CREA), and blood urea nitrogen (BUN). The panel of tests measuring energy metabolism included blood glucose (GLU), triglycerides (TG), cholesterol (CHOL), high-density lipoprotein (HDL), low-density lipoprotein (LDL). The panel of tests measuring body damages or inflammation included lactate dehydrogenase (LDH), creatine kinase (CK), high-sensitive C-reactive protein (hs-CRP).

### Gut microbiota analysis

#### DNA extraction and next generation sequencing (NGS)

Bacteria DNA extraction was performed on the fecal samples using the QIAamp^®^ DNA Mini Kit (QIAGEN Canada, Mississauga, ON, Canada), following the manufacturer’s protocol. After extraction and purification, the DNA was used as the polymerase chain reaction (PCR) template for amplification. The bacterial V3-V4 region of 16S rRNA was amplified using the specific primer pair 314F (5’-TCGTCGGCAGCGTCAGATGTGTATAAGAGACAGCCTACGGGNGGCWGCAG-3’) and 805R (5’-GTCTCGTGGGCTCGGAGATGTGTATAAGAGACAGGACTACHVGGGTATCTAATCC-3’) [33, 34]. The amplification was performed with KAPA HiFi HotStart ReadyMix [Roche Sequencing Solutions, Pleasanton, CA, USA (KK2601)] through the following steps: 95°C for 5 min; followed by 30 cycles of 95°C for 30 s, 60°C for 30 s, and 72°C for 30 s; and final extension at 72°C for 5 min. The PCR products were stored at 4°C and then used as templates for Index PCR, which was run under the following conditions: 95°C for 30 s; 8 cycles of 95°C for 30 s, 60°C for 30 s, and 72°C for 30 s; and final extension at 72°C for 5 min. DNA samples were paired-end sequenced (2 × 300 bp) on an Illumina MiSeq platform (Illumina, San Diego, USA) by Majorbio Bio-Pharm Technology Co., Ltd. (Shanghai, China).

#### Flora diversity and statistical analysis

Sequence data were performed using 16S Metagenomics apps on Basespace (Illumina, San Diego, CA, USA) and the reads were clustered to operational taxonomic units (OTUs) with Illumina-curated version of May 2013 Greengenes taxonomic database for downstream analysis. The Simpson index was used to indicate the alpha diversity of bacteria, in which a larger index represents higher community diversity. Beta diversity was estimated with Jaccard index using MicrobiomeAnalyst 2.0 [35], and principal coordinate analysis (PCoA) was calculated to analyze changes in species composition on time and space scales.

#### Heatmap of microbiota modulation and statistical analysis DNA extraction and next generation sequencing (NGS)

The percentage of each bacterium was calculated by dividing its individual hit number by the total hit number. The difference between the end and the beginning of the intervention was assessed using Student’s t-test. Statistical significance is denoted as the *P*-value.

### The THP-1-HepG2 cell system analysis

#### The co-culture supernatant of probiotic bacteria and THP-1-differentiated macrophages (SPT) preparation.

THP-1 monocytic leukemia cells (ATCC TIB-202) were inoculated in a 75T flask at a density of 2 × 10^5^ cells/ml and grown to a density of 10^7^ cells/ml. The THP-1 cells were differentiated into macrophages using a 24-hours phorbol-12-myristate-13-acetate (PMA, Sigma, United States) treatment. After resting in fresh medium for 24 hours, the THP-1 macrophages were detached from the culture plate by adding 5 ml 0.25% trypsin. Then, the THP-1-differentiated macrophages were seeded into a 12-well plate at a density of 8 × 10^5^ cells/ml/well, and co-cultured for 24 hours with probiotic bacteria at a cell to bacteria ratio of 1:50 after cell attachment. The supernatant was collected by centrifugation at 4000 rpm for 10 min and 0.22 μm filtration. The SPT was store at −20°C for further use. The supernatant of THP-1-differentiated macrophages served as a blank SPT.

#### Oil Red O staining in HepG2 cells

The free fatty acid (FFA) medium was prepared by adding 186 μL 354 mM oleic acid and 846 μL 39 mM palmitic acid in Dulbecco’s Modified Eagle Medium (DMEM, Cytiva, United States) medium containing 10% fetal bovine sera (FBS, Gibco, United States), 1% penicillin-streptomycin (PS, Cytiva, United States), 2% bovine serum albumin (BSA, EMD Millipore, United States). HepG2 human hepatoma cells (BCRC 60177) were seeded into a 6-well plate and incubated for 3 days before further treatments. Then, the HepG2 cells were treated with 1 mM FFA medium for 24 hours to induce hepatic steatosis [36]. After washing with phosphate-buffered saline (PBS) twice, the HepG2 cells were treated for 48 hours with either 1mM FFA medium, 1 μg/mL lipopolysaccharide (LPS, Sigma, United States) or 50 μL/mL SPT mentioned above. After washing with PBS twice, the HepG2 cells were fixed with 10% formalin, washed with 60% isopropanol, and stained with 0.5% oil red O (Sigma-Aldrich, United States). After the stained HepG2 cells were observed at 100x magnification under a microscope (Nikon, Japan), the oil red O dye was eluted by 100% isopropanol and measured at OD_520_ by a CLARIOstar^®^ Plus (BMGlabtech, Germany).

#### The uric acid excretion in Caco-2 cells

Caco-2 human colon carcinoma cells (BCRC 67001) were seeded and grown as a monolayer on a 0.4 μm Falcon^®^ permeable support (CORNING, United States). Before treatments, the trans-epithelial electric resistance (TEER) across the Caco-2 monolayer was measured with fresh DMEM medium by a EVOM2 Epithelial Voltohmmeter (World Precision Instruments, United States). Then, the Caco-2 cells were treated with DMEM medium containing probiotic bacteria in the upper compartment, and DMEM medium containing 10 μM UA in the lower compartment. The UA concentration in the upper compartment was measured at 30 minutes as the starting point and at 24 hours as the ending point with a UA assay kit (Cayman Chemical, United States) by a CLARIOstar^®^ Plus microplate reader (BMG LABTECH, Germany). After washing with PBS twice, the TEER across the Caco-2 monolayer was measure with fresh DMEM medium again at the end of the treatment.

#### Gene expression analysis

For cytokine gene expression (IL-6, IL-8 and CCL2), the HepG2 cells grown in a 6-well culture plate for 3 days, and then treated with 1 mM FFA medium for 24 hours. After washing with PBS twice, the HepG2 cells were treated for 48 hours with either 1 mM FFA medium or 50 μL/mL SPT mentioned above. Except for the FFA group, all groups were treated with 1 μg/mL LPS for further 3 hours, and cells were harvested for qPCR analysis.

For UA transporter gene expression (ABCG2 and SLC2A9), the Caco-2 cell was grown as a monolayer for 6 days on a 0.4 μm Falcon^®^ permeable support (CORNING, United States). The Caco-2 monolayer was treated for 6 hours with DMEM medium containing probiotic bacteria in the upper compartment, and DMEM medium containing 10 μM UA in the lower compartment. After removing medium in the upper compartment, Caco-2 cells were harvested for qPCR analysis.

RNA was extracted with Trizol (Invitrogen, United States). The cDNA was generated with a GoScript^™^ Reverse Transcriptase (Promega, United States), and qPCR was performed with a PB20.12–05 qPCRBio SyGreen Mix Hi-ROX (PCR Biosystems, United States) by an ABI StepOnePlus^™^ qPCR machine (Applied Biosystems, United States). The primer pair 5’-CATCCTCGACGGCATCTCAG-3’ and 5’-TGCCTCTTTGCTGCTTTCAC-3’ was used to detect IL-6 expression [37]. The primer pair 5’-CTGGCCGTGGCTCTCTTG-3’ and 5’-CCTTGGCAAAACTGCACCTT-3’ was used to detect IL-8 expression [36]. The primer pair 5’-CTCAGCCAGATGCAATCAATG-3’ and 5’-AGATCACAGCTTCTTTGGGACAC-3’ was used to detect CCL2 expression [36]. The primer pair 5’-AATACATCAGCGGATACTA-3’ and 5’-AATAAGCCACCATCATAAG-3’ was used to detect ABCG2 expression [38]. The primer pair 5’-CAATAGACCCAGACACTCTGACT-3’ and 5’-TCTTCACAATTAACGTCCCCAC-3’ was used to detect SLC2A9 expression [38]. The primer pair 5’-GAAGATGGTGATGGGATTTC-3’ and 5’-GAAGGTGAAGGTCGGAGT-3’ was used to detect GAPDH expression as the internal control [39].

#### Statistical analysis

One-way analysis of variance (ANOVA) was used to examine the differences among all groups, and Student’s t-test was used to compare the differences between two groups. The paired t-test was used to compare the difference before and after the intervention within each group. The difference of *P* < 0.05 was considered statistically significant. The change rate of the test item was presented as the standardized mean percentage (Value_End_/Value_Start_). Figs were generated by using Graphpad prism 8 (Graphpad Software, San Diego, CA, United States). The statistical analyses were performed utilizing SPSS software (IBM, Armonk, NY, USA).

## Results

**Fig 1:**
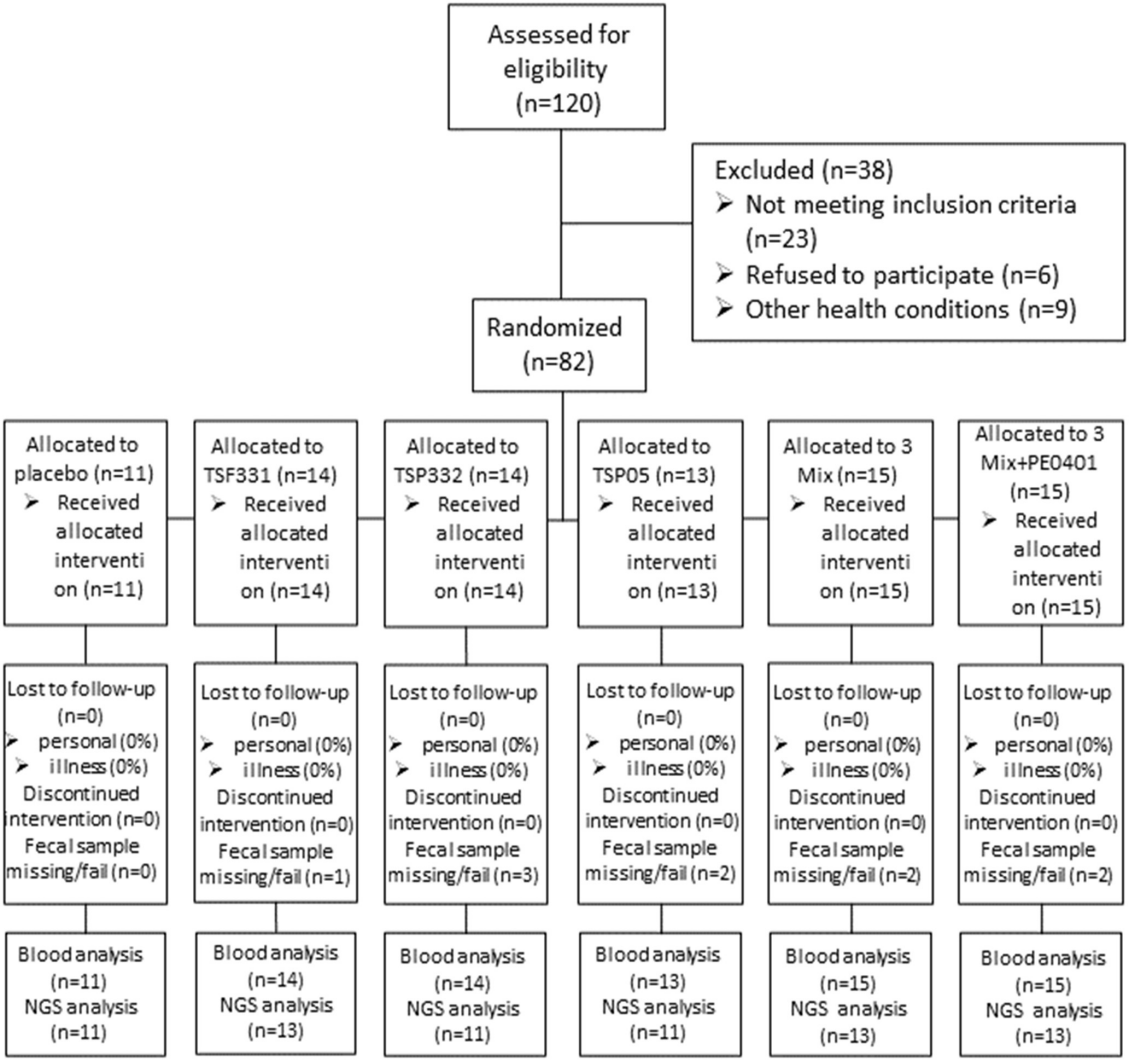
The subject enrollment, randomization, and disposition were presented in the diagram.

The clinical study was carried out as the flowchart in Fig 1.

One hundred and twenty participants were recruited according to their latest physical examination report. Twenty-three participants were excluded due to unqualified blood biochemistry profiles on day 0. Additionally, six participants were excluded because of improperly signed informed consents, while nine others were excluded due to other underlying health conditions. Eighty-two participants were randomized into 6 groups, and all subjects completed the intervention (11 in the placebo group, 41 in the mono-strain group, 15 in the 3 Mix groups, and 15 in the 3 Mix+PE0401 group). Ten fecal samples were excluded due to poor storage or bad sample quality. In the end, the blood chemistry was analyzed in 82 subjects, and gut microbiota was analyzed in 72 subjects.

### The multi-strain probiotics plus postbiotics enhanced the protective effect on liver health subsection

**Fig 2:**
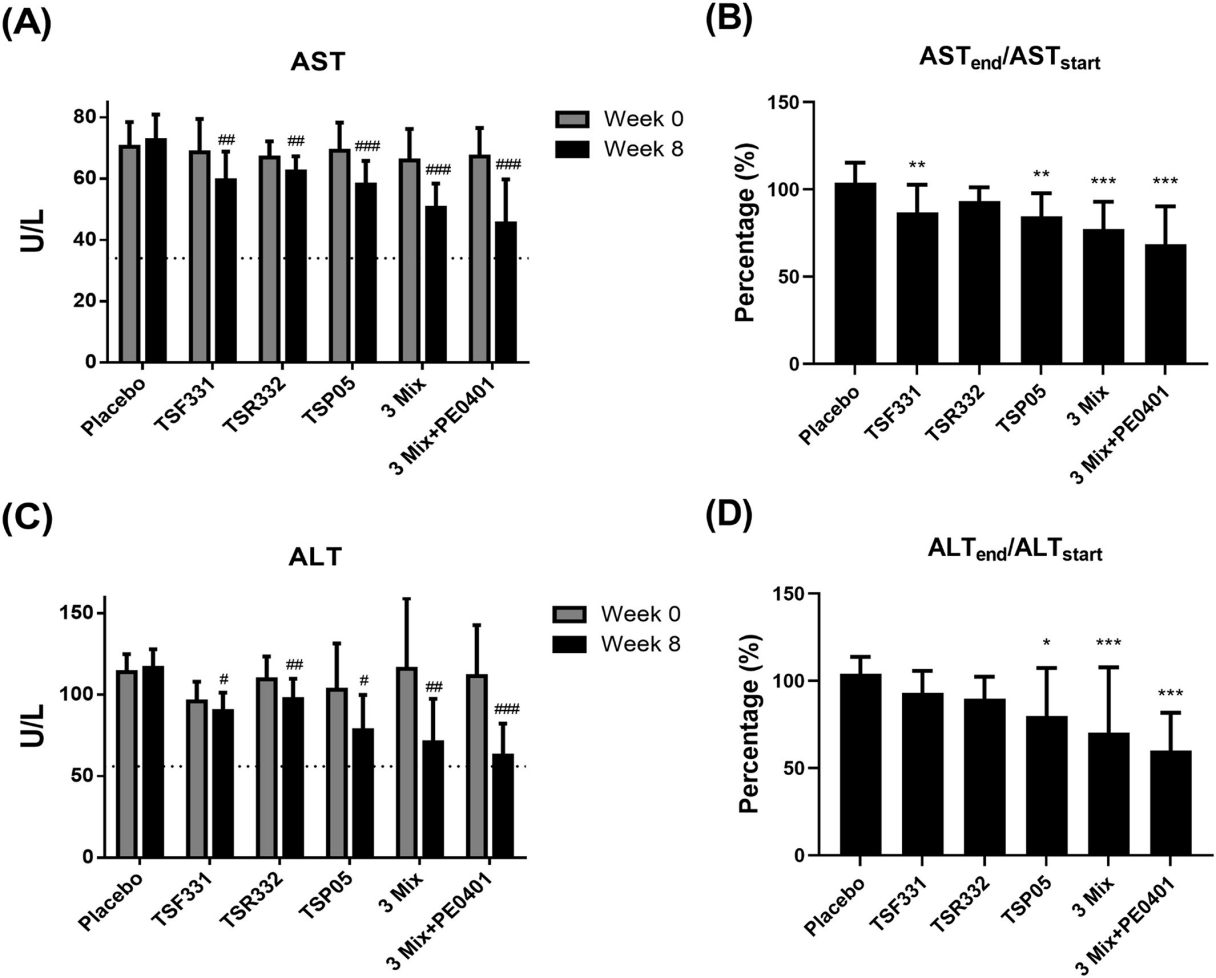
The effect of probiotic supplementation on liver health was evaluated in liver enzymes. (A) The AST levels were recorded on day 0 and 60. (B) The difference of serum AST levels between before and after the intervention was converted to the change rate in each group. (C) The serum ALT levels were recorded on day 0 and 60. (D) The difference of serum ALT levels between before and after the intervention was converted to the change rate in each group. The dotted lines denote the upper limit of normal serum AST and ALT levels. Data are presented as mean ± standard deviation (SD) of the results from each subject. ^#^The paired t-test was used to compare the difference before and after the intervention within each group, ^#^*P* < 0.05, ^##^*P* < 0.01, ^###^*P* < 0.001. The one-way analysis of variance (ANOVA) was utilized to compare the change rate with the control group, followed by the least significant difference (LSD) post hoc test for subsequent analysis. Statistical significance was denoted as *P < 0.05, **P < 0.01, and ***P < 0.001.

To assess the impact of the intervention on liver health, a comprehensive panel of tests measuring liver function was conducted using blood samples obtained from the subjects. Before the initiation of the intervention on day 0, there was no significant difference in the serum AST levels among the groups (*P* = 0.735, Fig 2A). After the intervention on day 60, a notable disparity in the serum AST levels emerged among the groups (*P* < 0.001, Fig 2A). All treatment groups demonstrated a marked reduction in serum AST levels compared to the control group (*P* < 0.05). Remarkably, within each treatment group, serum AST levels exhibited a significant reduction compared to their respective baseline levels (*P* < 0.01). The placebo group showed a marginal increase of 3.84 ± 11.53%. In contrast, with the exception of the TSR332 group, the TSF331, TSP05, 3 Mix, and 3 Mix+PE0401 groups demonstrated substantial reductions of 13.09 ± 15.82%, 15.46 ± 13.29%, 22.61 ± 15.57%, and 31.43 ± 21.69%, respectively (Fig 2B). Particularly noteworthy is the multi-probiotics plus postbiotics group, which exhibited the most significant reduction in AST levels among all the groups.

Similarly, there was no discernible difference in the serum ALT levels among the groups prior to the initiation of the intervention on day 0 (*P* = 0.683, Fig 2C). A notable divergence in the serum ALT levels emerged among the groups, showing a significant contrast post-intervention on day 60 (*P* < 0.001, Fig 2C). Notably, within each treatment group, serum ALT levels showed a substantial reduction compared to their respective baseline levels (*P* < 0.05). The placebo group experienced a marginal increase of 4.01 ± 9.68%. In contrast, with the exception of the TSF331 and TSR332 groups, the TSP05, 3 Mix, and 3 Mix+PE0401 groups demonstrated noteworthy reductions of 20.26 ± 27.59%, 29.74 ± 37.43%, 40.00 ± 21.72%, respectively (Fig 2D). Particularly remarkable is the multi-probiotics plus postbiotics group, showcasing the most significant reduction in ALT levels among all the groups.

### The multi-strain probiotics plus postbiotics enhanced the UA lowing effect

**Fig 3:**
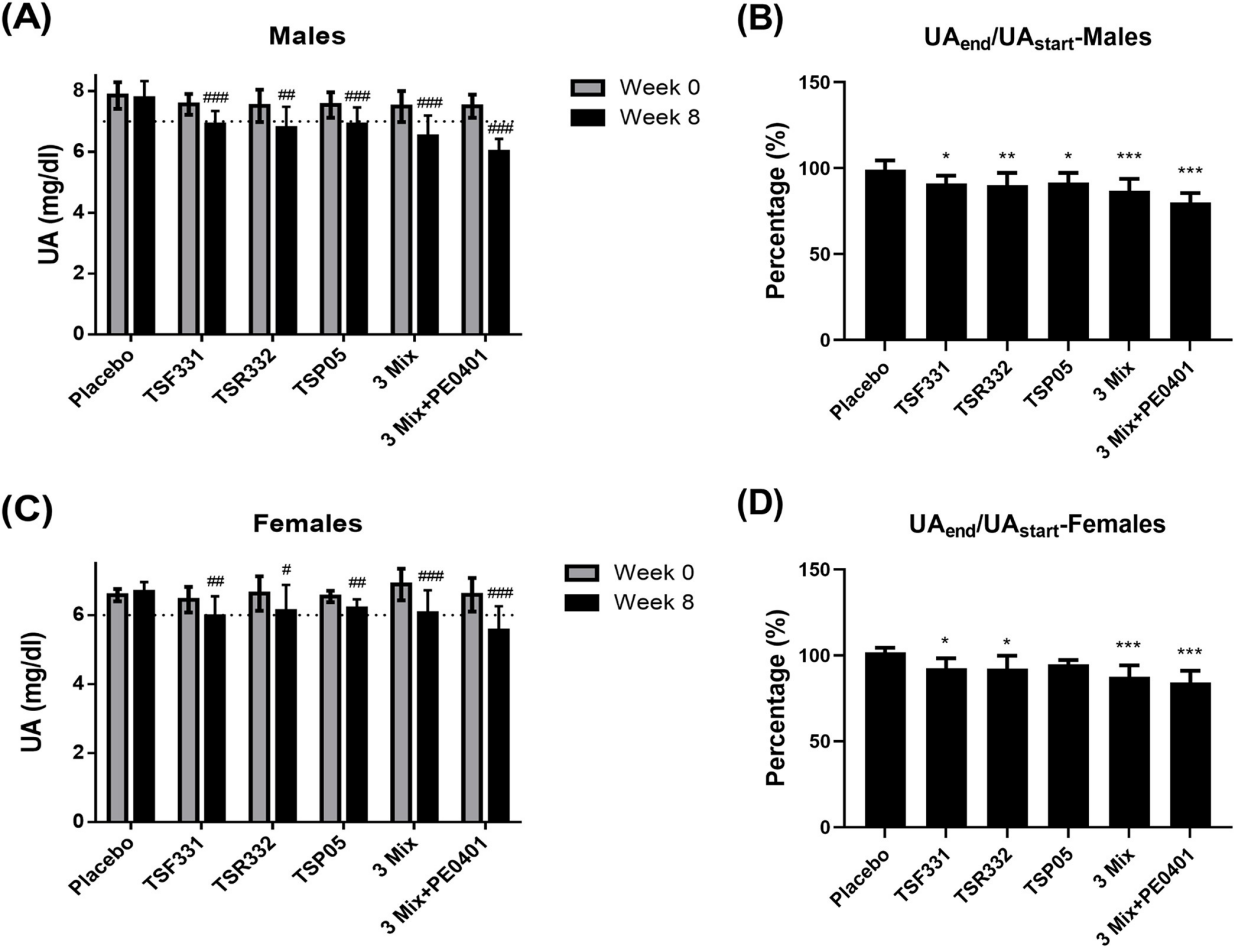
The effect of probiotic supplementation on UA management was investigated in mono- and multi-strain groups. (A) The serum UA levels were recorded in male on day 0 and 60. (B) The difference of serum UA levels between before and after the intervention was converted to the change rate in male subjects in each group. (C) The serum UA levels were recorded in female on day 0 and 60. (D) The difference of serum UA levels between before and after the intervention was converted to the change rate in female subjects in each group. The dotted lines denote the upper limit of normal serum UA levels. Data are presented as mean ± SD of the results from each subject. ^#^ The paired t-test was used to compare the difference before and after the intervention within each group, ^#^*P* < 0.05, ^##^*P* < 0.01, ^###^*P* < 0.001. The one-way ANOVA was utilized to compare the change rate with the control group, followed by the LSD post hoc test for subsequent analysis. Statistical significance was denoted as *P < 0.05, **P < 0.01, and ***P < 0.001.

UA levels can vary between males and females, partly due to the sex hormone. To assess the impact of the intervention on UA management, the serum UA levels were separately analyzed in the male and female subjects. In males, there was no significant difference in the serum UA levels among the groups before the intervention on day 0 (*P* = 0.535, Fig 3A). After the intervention on day 60, a significant difference in the serum UA levels appeared among the groups (*P* < 0.001, Fig 3A). All treatment groups demonstrated a marked reduction in serum UA levels compared to the control group (*P* < 0.01). Notably, within each treatment group, serum UA levels exhibited a significant reduction compared to their respective baseline levels (*P* < 0.01). The placebo group showed a marginal reduction of 0.97 ± 5.59%, while the TSF331, TSR332, TSP05, 3 Mix, and 3 Mix+PE0401 groups demonstrated substantial reductions of 8.77 ± 4.44%, 9.76 ± 7.04%, 8.29 ± 5.61%, 13.01 ± 6.81%, 19.93 ± 5.43%, respectively (Fig 3B). The serum UA levels for males were significantly reduced in all probiotic groups and, remarkably, the greatest reduction was observed in the multi-probiotics plus postbiotics group.

In females, no discernible difference was observed in the serum UA levels among the groups prior to the initiation of the intervention on day 0 (*P* = 0.793, Fig 3C). There was no statistical significance in the serum UA levels was detected among the groups after the intervention on day 60 (*P* = 0.188, Fig 3C). Remarkably, within each treatment group, serum UA levels underwent a notable reduction compared to their respective baseline levels (*P* < 0.05). The placebo group displayed a slight increase of 1.69 ± 2.79%, while the TSF331, TSR332, 3 Mix, and 3 Mix+PE0401 groups exhibited significant reduction of 7.47 ± 5.77%, 7.77 ± 7.58%, 12.46 ± 6.62%, 15.76 ± 6.81%, respectively (Fig 3D). The serum UA levels for females were significantly reduced in all probiotic groups, with the exception of the TSP05 group. Notably, the greatest reduction was observed in the multi-probiotics plus postbiotics group.

### The multi-strain probiotics plus postbiotics enhanced the improvement on energy metabolism

Despite the wide age range among the subjects, the allocation was evenly distributed (*P* = 0.161, S1 Table). The blood chemistry profiles were initially comparable, with measurements, e.g., TBIL, GGT, and ALKP, in the liver function panel falling within the normal range across all groups on day 0 (S1 Table). Remarkably, by day 60, the blood chemistry profiles continued to remain within the normal range (Table 1).

**Table 1:** The blood biochemical profile on day 60.

	Placebo (N = 11)	TSF331 (N = 14)	TSR332 (N = 14)	TSP05 (N = 13)	3 Mix (N = 15)	3 Mix +PE0401 (N = 15)	*P*-value
**TBIL**	0.82	0.58	0.82	0.80	0.59^#^	0.81^#^	0.136
**(mg/dL)**	± 0.35	± 0.26	± 0.32	± 0.36	± 0.25	± 0.40	
**GGT**	23.18	29.43	27.07	27.85	23.13^###^	20.40*^###^	0.394
**(U/L)**	± 13.28	± 16.35	± 10.99	± 5.87	± 6.51	± 17.72	
**ALKP**	54.36	62.00	58.21	63.23	51.33	52.00^##^	0.358
**(U/L)**	± 7.57	± 13.52	± 24.18	± 20.82	± 15.57	± 19.54	
**ALB**	4.46^#^	4.83	4.51	4.57	4.41	4.41	0.076
**(g/dL)**	± 0.24	± 0.48	± 0.39	±0.42	± 0.22	± 0.22	
**CREA**	0.85	0.85	0.75	0.77	0.74	0.74	0.096
**(mg/dL)**	± 0.10	± 0.14	± 0.14	± 0.11	± 0.14	± 0.14	
**BUN**	13.55	14.61	13.61	15.62	11.92	11.92	0.466
**(mg/dL)**	± 5.40	± 6.26	± 4.24	± 6.83	± 2.60	± 2.60	
**GLU**	88.91	80.36*^#^	77.79*^#^	77.92*^#^	78.27*^#^	78.73*^#^	0.206
**(mg/dL)**	± 7.08	± 12.11	± 10.42	± 12.44	± 15.33	± 11.98	
**TG**	124.55	110.64	116.79^#^	111.92^##^	104.33*^##^	102.20*^###^	0.256
**(mg/dL)**	± 39.06	± 15.00	± 22.11	± 20.72	± 31.97	± 18.92	
**CHOL**	183.82	163.29	176.29	160.08^#^	160.27^#^	160.80*^##^	0.341
**(mg/dL)**	± 25.98	± 38.42	± 37.99	± 33.53	± 31.13	± 29.99	
**LDL**	115.25	100.64	84.16	99.13	90.59	95.31^#^	0.295
**(mg/dL)**	± 30.80	± 29.72	± 34.88	± 36.48	± 30.33	± 35.89	
**HDL**	51.55	54.61	53.23	51.91	49.72	51.36	0.909
**(mg/dL)**	± 12.09	± 12.17	± 10.53	± 13.72	± 10.00	± 10.90	
**Hs-CRP**	0.19^a^	0.26^a^	0.32^b^	0.27^a^	0.15^a^	0.19^a^	0.042
**(mg/dL)**	± 0.08	± 0.18	± 0.16	± 0.15	± 0.14	± 0.17	
**LDH**	109.27	123.43	97.93	110.62	99.07	89.00**^##^	0.058
**(U/L)**	± 28.99	± 32.52	± 30.62	± 36.50	± 32.12	± 19.78	
**CK**	112.55	111.29	102.71	80.69	81.67	98.27	0.362
**(U/L)**	± 65.94	± 52.05	± 44.59	± 40.27	± 36.22	± 50.05	

Data are presented as mean ± SD of the results from each subject. The *P*-value presented the difference among groups using One-way ANOVA. ^a,b^ Groups with different letters are considered significantly different from each other, *P* < 0.05. Change rate was calculated as value_end_/value_start_ × 100% in the same subject. * The change rate was compared between placebo and treatment groups using Student’s t-test, *P < 0.05, and **P < 0.01. ^#^ The difference before and after the intervention was compared within each group using a paired t-test, ^#^*P* < 0.05, ^##^*P* < 0.01, ^###^*P* < 0.001.

Notably, the probiotic intervention exerted discernible effects on various energy metabolic panels. Specifically, the serum glucose (GLU) levels exhibited a reduction in all probiotic groups. Additionally, the blood lipid profile, including TG, CHOL, and LDL, showed a decrease in the multi-probiotics plus postbiotics group.

### Probiotic supplementation modulated gut microbiota without affecting diversity

**Fig 4:**
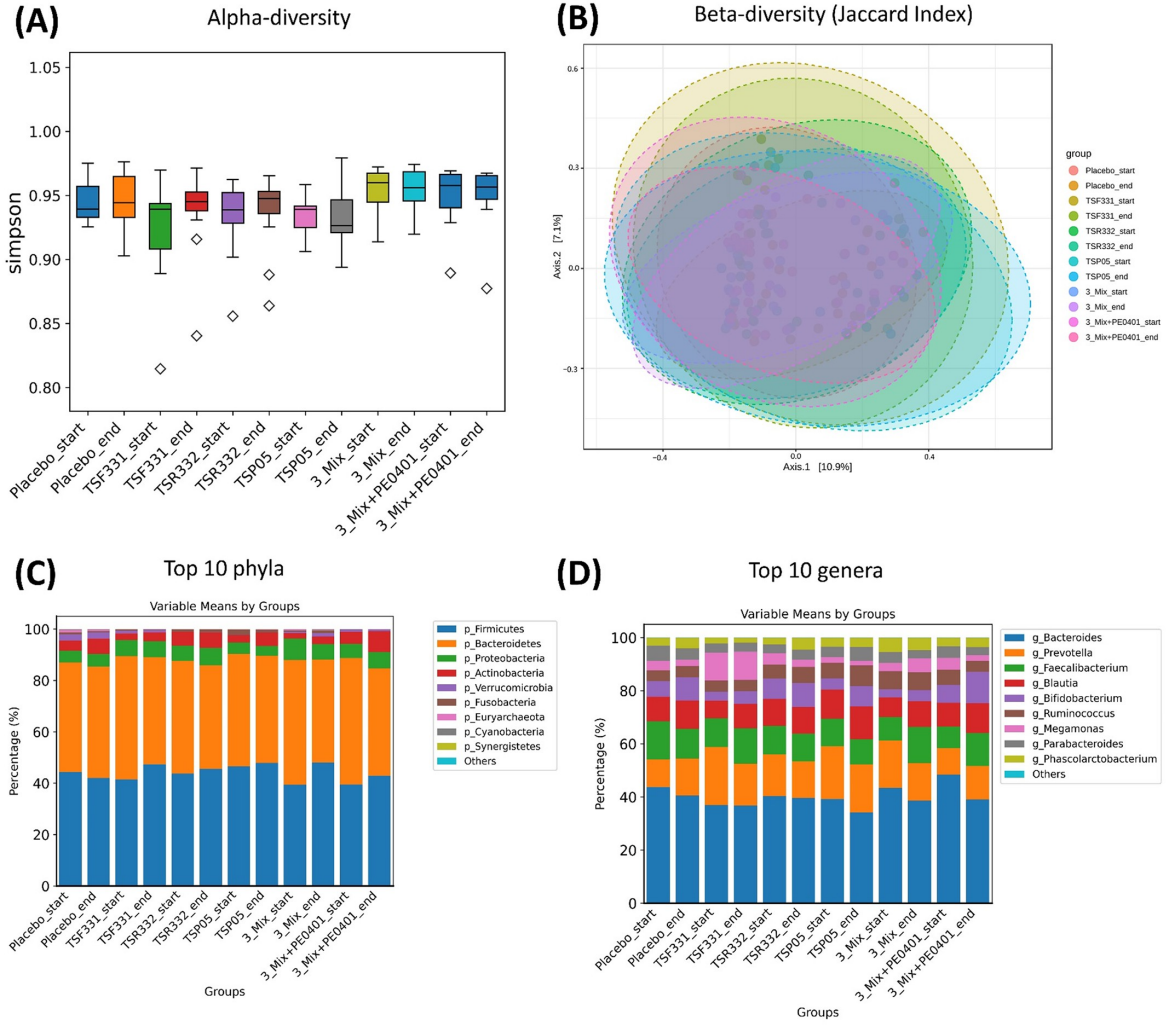
The effect of probiotic supplementation on gut microbiota was analyzed by NGS. No significant alteration was detected in (A) alpha diversity and (B) beta diversity. The composition of the 10 most abundant (C) phyla and (D) genera was not affected by the intervention in all groups.

To assess the modulation of gut microbiota through probiotic supplementation, the microbial composition in fecal samples was compared between day 0 and day 60. No significant alteration was detected in alpha and beta diversity (Fig 4A and 4B). Two phyla Firmicutes and Bacteroidetes composed more than 80% of the flora (Fig 4C). Five genera *Bacteroides*, *Prevotella*, *Faecalibacterium*, *Blautia*, and *Bifidobacterium* represented more than 80% of the population (Fig 4D). Prior to the intervention, the patterns of the 10 most abundant phyla and genera were similar but not identical among the groups, and the probiotic supplementation did not dramatically alter these patterns in any of the groups.

**Fig 5:**
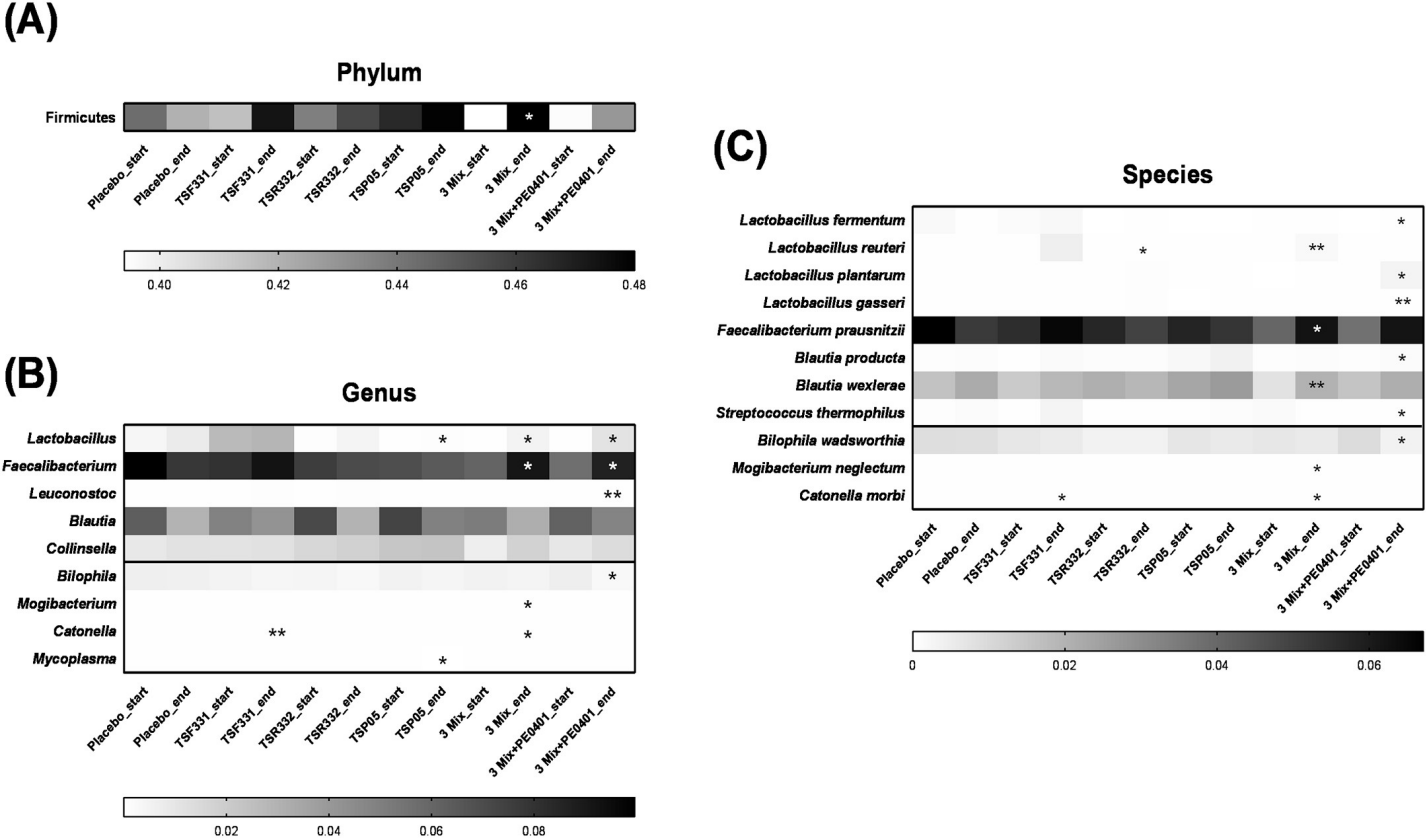
The probiotic supplementation promoted the growth of commensal bacteria and inhibited pathobiont bacteria in gut microbiota. The intervention resulted in significant alterations to the abundance of (A) one phylum, (B) seven genera, and (C) eleven species. * The paired t-test was employed to assess the differences in abundance within the group before and after the intervention, *P < 0.05, and **P < 0.01.

While preserving diversity, several microbial changes occurred. The abundance of the Firmicutes phylum significantly increased in the 3 Mix group (Fig 5A). Notably, the 3 Mix and 3 Mix+PE0401 groups displayed the best efficiency on the modulation. Two probiotic genera (*Lactobacillus* and *Faecalibacterium*) showed significant increases in the 3 Mix group, while two pathobiont genera (*Mogibacterium* and *Catonella*) decreased (Fig 5B). Three probiotic genera (*Lactobacillus*, *Faecalibacterium*, and *Leuconostoc*) showed significant increases in the 3 Mix+PE0401 group, while one pathobiont genus, *Bilophila*, decreased (Fig 5B). Additionally, three probiotic species (*L*. *reuteri*, *F*. *prausnitzii*, and *B*. *wexlerae*) significantly increased, and two pathobiont species (*M*. *neglectum* and *C*. *morbi)* decreased in the 3 Mix group (Fig 5C). Five probiotic species (*L*. *fermentum*, *L*. *plantarum*, *L*. *gasseri*, *B*. *producta*, and *S*. *thermophilus*) significantly increased, and one pathobiont species, *B*. *wadsworthia*, decreased in the 3 Mix+PE0401 group (Fig 5C).

### *Lactobacillus* strains reduced the lipid accumulation and inflammatory factor expressions in a gut-liver axis system

**Fig 6:**
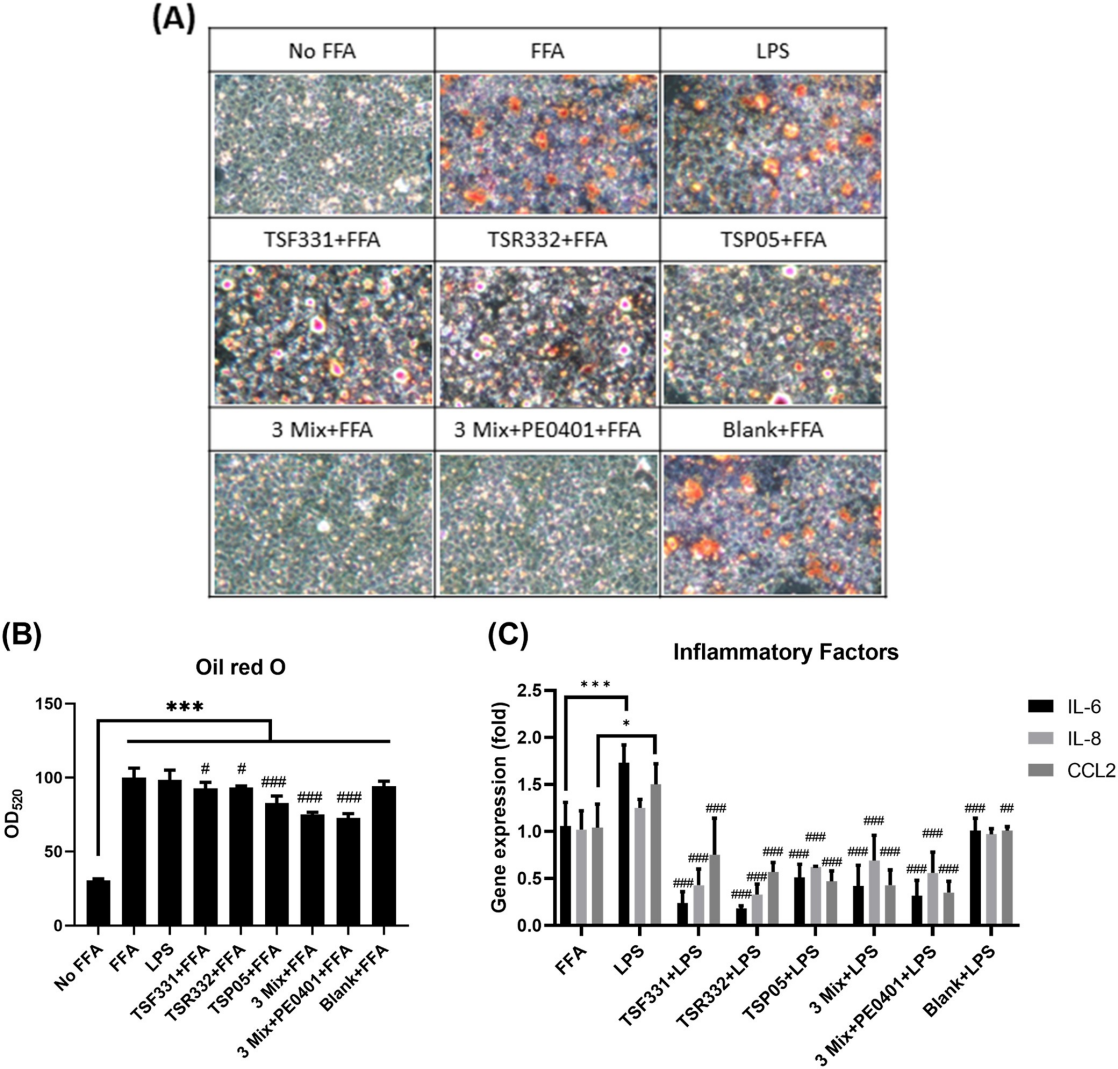
The SPT treatments reduced lipid droplet accumulation and inflammatory factor expression in HepG2 cells. (A) The lipid droplet accumulation in HepG2 cells was observed at 100x magnification under microscope. (B) The oil red O dye was eluted and measured at OD_520_. Data were analyzed using one-way ANOVA, followed by the LSD post hoc test. Significance levels were indicated as follows: ***P < 0.001 compared to No FFA; ^#^*P* < 0.05, and ^###^*P* < 0.001 compared to FFA. (C) The gene expression of IL-6, IL-8, and CCL2 was analyzed by RT-qPCR. Blank: The supernatant of THP-1-differentiated macrophages without co-culturing with probiotic bacteria. Data were analyzed using one-way ANOVA, followed by the LSD post hoc test. Significance levels were denoted as follows: for the difference between FFA and LPS groups, *P < 0.05 and ***P < 0.001; for the difference between LPS and SPT treatment groups, ^##^*P* < 0.01 and ^###^*P* < 0.001. All experiments were performed in triplicate and data are presented as mean ± SD.

To ascertain whether the oral probiotic supplementation reduced serum AST and ALT levels by mitigating lipid accumulation and inflammation, the oil red O staining and the expression of inflammatory factor genes were conducted in a THP-1-HepG2 cell system. The oil red O staining reveled a red color for the lipid accumulation in both FFA and LPS treated HepG2 cells (Fig 6A). The lipid accumulation was quantified by eluting the cellular oil red O dye, and the dye concentration was significantly reduced in all treatment groups (Fig 6B). The LPS treatment significantly elevated the expression of inflammatory factors IL-6 and chemokine (C-C motif) ligand 2 (CCL2) in FFA-treated HepG2 cells. Conversely, the expression of IL-6, IL-8, and CCL2 was significantly reduced in all SPT treatment groups (Fig 6C).

### *Lactobacillus* strains enhanced UA transporter expressions and heightened the UA excretion in Caco-2 cells

**Fig 7:**
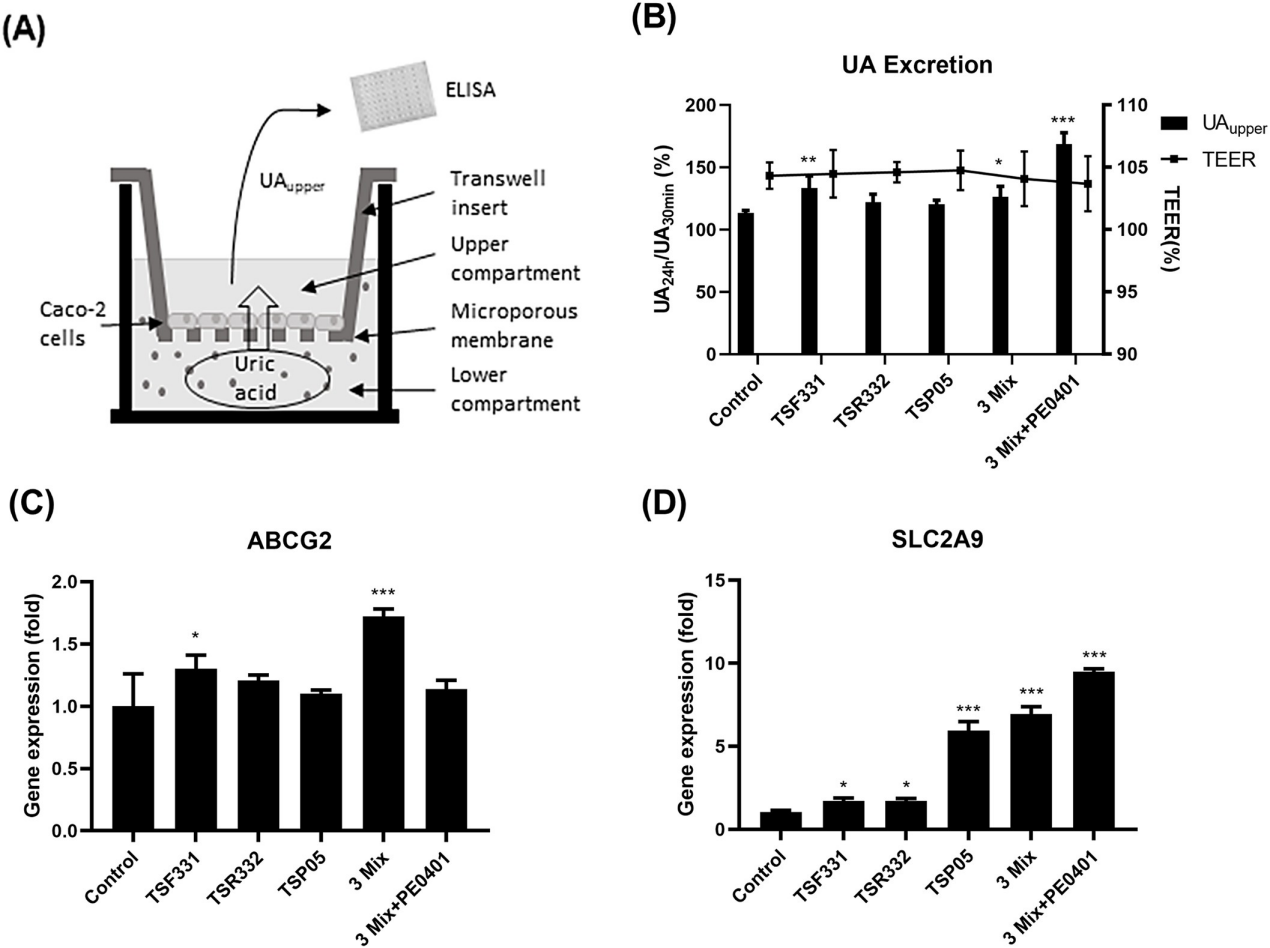
The probiotic bacteria promoted UA excretion and the gene expression of UA transporters in Caco-2 cells. (A) UA was introduced into the lower compartment, and the concentration of UA in the upper compartment was analyzed using enzyme-linked immunosorbent assay (ELISA). (B) The UA concentration in the upper compartment was recorder at 30 min and 24 hours. At the end of 24 hours incubation, the TEER was recorded to exam the integrity of Caco-2 monolayer. The gene expression of UA transporters, (C) ABCG2 and (D) SLC2A9, was analyzed by RT-qPCR. All experiments were performed in triplicate and data are presented as mean ± SD. Data were analyzed using one-way ANOVA, followed by the LSD post hoc test. Significance levels were indicated as follows: *P < 0.05, **P < 0.01, and ***P < 0.001 compared to control.

To confirm whether oral probiotic supplementation reduces serum UA levels by enhancing UA excretion in the gastrointestinal tract, the directional UA transport experiments were conducted using the Transwell system as indicated in Fig 7A. In the presence of a well-maintained Caco-2 monolayer with good integrity, efficient transport of UA from the lower compartment to the upper compartment was observed in the TSF331, 3 Mix, and 3 Mix+PE0401 groups (Fig 7B). ABCG2 is a membrane transporter involving in the efflux of UA from intestinal cells, and its expression was enhanced in the TSF331 and 3 Mix groups (Fig 7C). SLC2A9 is another transporter playing a role in the regulation of UA levels in the body, and its expression significantly increased in all treatment groups (Fig 7D).

## Discussion

This pilot study sought to assess the varying efficacy of mono-strain, multi-strain, and multi-strain plus postbiotics in the individuals at high risk of MAFLD. The findings should be interpreted cautiously owing to the limited sample size within each group. Nonetheless, our results indicate that further investigation into the efficacy of multi-strain and multi-strain plus postbiotics is warranted. In the context of treating irritable bowel syndrome (IBS), a systematic review revealed more pronounced beneficial effects in trials utilizing multi-strain supplements as opposed to mono-strain alternatives [40]. The gut microbiota is a microbial ecosystem containing a complex and diverse community of microorganisms. Gut microbiota dysbiosis is disruptions in the balance, which may lead to neurodegenerative diseases, cardiovascular diseases, metabolic diseases and gastrointestinal diseases [41]. Hence, the synergistic effect of combining probiotics and postbiotics may be more effective than mono-strain probiotics in creating a harmonious environment within the gut, resulting in enhanced health benefits.

As of now, the information establishing a clear relationship between a specific genus or species and metabolic disturbances remains limited. Our study sheds the light on a potential target for reshaping the gut microbiota. Particularly in the 3 Mix+PE0401 group, we observed a significant inhibition of the growth of *Bilophila*, especially the pathobiont *B*. *wadsworthia*. In addition, several animal investigations have indicated an increase in the abundance of *Bilophila* within the gut microbiota of high-fat diet (HFD)-fed rats, implying an association between this bacterial genus and high-fat dietary intake [42, 43]. Enrichment of *B*. *wadsworthia* in the host’s intestinal tract may precipitate disruptions in bile acid metabolism, inflammation, and compromise intestinal barrier integrity. Consequently, these disturbances may exacerbate glucose metabolism disorders and hepatic steatosis [43–45]. Totipro^®^ PE0401 is postbiotics consisted of metabolites from *Lactobacillus salivarius* AP-32, *L*. *acidophilus* TYCA06, *L*. *plantarum* LPL28, *Bifidobacterium longum* subsp. *infantis* BLI-02. Totipro^®^ PE0401 displayed the unique synergistic effect on the expression of tight junction proteins (TJPs) and the growth promoting effect on various probiotic bacteria strains [32]. Therefore, Totipro^®^ PE0401 may be able to enhance the health effect of probiotic bacteria via promoting intestinal barrier and beneficial bacteria growth. While some weak inhibition was observed in other treatment groups, the effect was not profound. Instead, there was a reduction in the abundance of genera *Mogibacterium*, *Catonella*, and *Mycoplasma* in certain groups. *Mogibacterium* exhibited a positive correlation with the pro-inflammatory factors interferon-γ and tumor necrosis factor-α in individuals diagnosed with medication-related osteonecrosis of the jaw (MRONJ) [46]. *Catonella* bacteria typically inhabit the gastrointestinal tract, but the presence of this genus, especially *C*. *morbi* species, in the oral cavity is associated with oral microbial dysbiosis [47, 48]. *Mycoplasma*s have a unique cell membrane and lack a rigid cell wall [49]. Although not all *Mycoplasma* species are naturally pathobionts, some commensals can become opportunistic pathobionts under certain conditions, especially when the host’s immune system is compromised [50]. In this study, varying degrees of growth inhibition were observed among pathobiont populations in different combinations of probiotics and postbiotics. The findings indicated that the combination of multi-strain probiotics with postbiotics exhibited the most effective inhibition of *B*. *wadsworthia*, a bacterium associated with glucose dysmetabolism and hepatic steatosis.

Lipid accumulation in the liver, also known as hepatic steatosis, occurs when there is an excessive build-up of fats (lipids), primarily TG, within liver cells (hepatocytes) [51]. In some cases, hepatic steatosis can progress to non-alcoholic steatohepatitis, which is characterized by not only fat accumulation but also liver inflammation and damage. Elevated levels of ALT and AST in the blood can be indicative of liver damage. Recently, accumulating data indicated that FFAs played a damaging role on liver cells and involved in the cross-talk between the gut and the liver [52]. A meta-analysis of randomized controlled trials concluded microbial therapies could improve liver steatosis and ALT levels [53]. Furthermore, an additional systematic review, encompassing 10 studies, assessed the influence of probiotics on liver function tests, demonstrating a beneficial effect of probiotics on ALT, AST, and GGT levels among patients with NAFLD [54]. In an animal study, feeding mice a HFD along with *L*. *paracasei* CCFM1224-derived postbiotics (800 mg/kg/day) was found to mitigate weight gain [55]. Simultaneously, it inhibited the accumulation of epididymal white adipose tissue and insulin resistance, improved serum biochemical indicators related to lipid metabolism, and alleviated hepatic steatosis and inflammation. The postbiotics were capable of modulating the gut microbiota of HFD-fed mice, thereby increasing the relative abundance of *Akkermansia*. Remarkably, our study demonstrated the combination of probiotic microbials (*L*. *fermentum* TSF331, *L*. *reuteri* TSR332, and *L*. *plantarum* TSP05) and postbiotics (Totipro^®^ PE0401) exhibited more effective ALT and AST reduction than the probiotics alone. This combination also displayed the lowest lipid accumulation and the enhanced anti-inflammatory effect *in vitro*. Symbiotic metabolites can modulate the composition and activity of the gut microbiota, promoting a balanced and diverse microbial community [56]. Moreover, symbiotic metabolites have been shown to possess anti-inflammatory properties, helping to mitigate excessive immune responses and reduce inflammation in the body [57]. Our study uncovered that the beneficial effects of combining probiotics and postbiotics are additive.

Presently, there are safety and tolerability concerns associated with urate-lowering drugs [58]. Drugs aimed at promoting UA excretion primarily target the kidneys, posing a heightened risk for kidney burden, particularly in chronic kidney disease patients. Hence, there is a potential shift towards the intestine as a safer and more effective target organ for urate-lowering drugs due to its substantial excretory capacity [59]. Remodeling the gut microbiota has been suggested as a promising therapeutic strategy to manage hyperuricemia and gout [60]. An animal study revealed that oral administration of *Lacticaseibacillus paracasei* MJM60396 (3 × 10^9^ cfu/mice/day) for three weeks significantly reduced serum uric acid levels to within normal range [61]. This effect was achieved through the inhibition of xanthine oxidase (XO) to decrease UA synthesis, as well as the modulation of uric acid transporters to enhance UA excretion. Another animal study showed that mice with hyperuricemia experienced a notable 35.5% decrease in serum uric acid concentration after consuming *Lactiplantibacillus plantarum* X7022 for four weeks [62]. Concurrently, levels of propionate and butyrate in their feces were elevated. These physiological changes could be attributed to the suppression of XO activity and the regulation of UA transport protein expression to normal levels. Furthermore, probiotics also ameliorated dysbiosis in the gut microbiota of hyperuricemia mice and promoted the production of SCFA-related microbiota. The supplementation of probiotic microbials contributed to the nucleoside degeneration in the gastrointestinal tract and the UA excretion in the feces and urine [63–66]. Interestingly, the combination of probiotic microbials (*L*. *fermentum* TSF331, *L*. *reuteri* TSR332, and *L*. *plantarum* TSP05) and postbiotics (Totipro^®^ PE0401) displayed more effective UA reduction compared to probiotics alone in our study. This combination exhibited the best UA excretion and UA transporter SLC2A9 upregulation in Caco-2 cells. However, the regulation may not be synchronized in different UA transporter genes, such as ABCG2. Further investigations are needed to illustrate the UA lowering mechanism by the combined action of probiotics and postbiotics in details.

Three *Lactobacillus* strains were introduced in this study, and several microbial changes in respond to the intervention. The rise in abundance within the phylum Firmicutes, as well as in the genera *Lactobacillus* and the specific species *L*. *fermentum*, *L*. *reuteri*, and *L*. *plantarum*, signifies the successful implementation of the intervention. Notably, the genera *Faecalibacterium* and *Leuconostoc* increased in the 3 Mix and 3 Mix+PE0401 groups. Low levels of *Faecalibacterium* spp. are reported to correlate with inflammatory conditions, and numerous studies have demonstrated the important role of *Faecalibacterium prausnitzii* in human health [67–70]. The *Leuconostoc* spp. are commonly used as starter bacteria in some dairy fermentations, and members in this genus were found to improve UA and liver metabolism in animals [71, 72]. While the overall increase in the *Blautia* genus did not reach statistical significance, noteworthy elevations were observed in the specific species *B*. *producta* within the 3 Mix+PE0401 group and *B*. *wexlerae* within the 3 Mix group. Recently, a substantial body of research has concentrated on exploring the probiotic effects of the *Blautia* genus, particularly its potential in alleviating metabolic syndrome [73]. *B*. *wexlerae* demonstrated efficacy in improving both obesity and type 2 diabetes [74], while *B*. *producta* emerged as a promising candidate for probiotic use in preventing acute liver injury [75]. Overall, our results indicated the multi-strain probiotics together with postbiotics was able to elevate commensal populations in the gut microbiota.

## Conclusions

The supplementation of multi-strain probiotics (*L*. *fermentum* TSF331, *L*. *reuteri* TSR332, and *L*. *plantarum* TSP05) together was able to prevent fatty liver by reducing lipid accumulation and inflammation. This combination was also more efficient on UA management by promoting UA excretion in the gut. The synergy between probiotics and postbiotics has the potential to establish a harmonious environment, specifically reshaping dysbiosis in metabolic disturbances.

## Supporting information

S1 Checklist CONSORT checklist. (DOC)

S1 Protocol Chinese protocol. (PDF)

S2 Protocol English protocol. (PDF)

S1 Table The blood biochemical profile on day 0. (DOCX)

S1 Data (XLSX)
