# Supplementary material for: Probiotic Lactobacillus fermentum TSF331, Lactobacillus reuteri TSR332, and Lactobacillus plantarum TSP05 improved liver function and uric acid management-A pilot study
Source: PLoS One. 2024 Jul 24;19(7):e0307181. doi: 10.1371/journal.pone.0307181 (PMC11268587; doi:10.1371/journal.pone.0307181)
Supplement: S1 Protocol — (PDF) [file pone.0307181.s002.pdf]

試驗計劃書

摘要:

腸道菌相對人類健康之影響，受到全球各界的重視與期待，益生菌更是消費者認知度最高的保健素材之一。近年國內消費者最常購買的保健食品主要原料，乳酸菌/益生菌位居第一，其功效為改善腸胃改善、免疫調節、調節血脂等。隨著人類文化的進步與生活習慣的變遷，許多現代文明病相繼而出，而這些疾病多半是因為生活作息或飲食的改變，造成人體的代謝失衡，如高血糖、高血脂、高血壓、高尿酸血症、肝功能失調及人體代謝失調所引起的肥胖等。腸道失衡與老化相關的疾病，通常會有致病菌增加與益生菌減少及菌叢多樣性下降的現象。菌相失衡會以產生脂多醣(LPS)、降低短鏈脂肪酸(SCFAs)、老化發炎(inflammaging)三個面向來嚴重影響人體代謝。次世代定序(NGS)技術，可了解人體腸道內菌相改善狀況。本研究目的為透過輔英老年暨疾病防治研究中心的臨床試驗，驗證代謝調節益生菌(*Lactobacillus fermentum* TSF331、*L. reuteri* TSR332、*L. plantarum* TSP05)在人體生化分析及人體腸道菌相的改善程度。透過本計畫，將會從臨床驗證的數個方向，包括血液生化數值及 NGS 腸道菌相檢測，檢測服用益生菌前後差異與改善狀況。

關鍵字: 腸道菌相、益生菌、代謝失衡、短鏈脂肪酸

前言:

代謝症候群(metabolic syndrome)

代謝症候群是一群容易導致心血管疾病的危險因子的總稱，這些危險因子主要包括高血壓、血脂異常(dyslipidemia)、糖尿病、肥胖、以及高尿酸與凝血因子的不正常等等。美國膽固醇教育計畫 III (The Third Report of the National Cholesterol Education Program Expert Panel，NCEP ATP III)對代謝症候群的定義是，滿足下列症狀標準(含)三項以上者，即為代謝症候群。

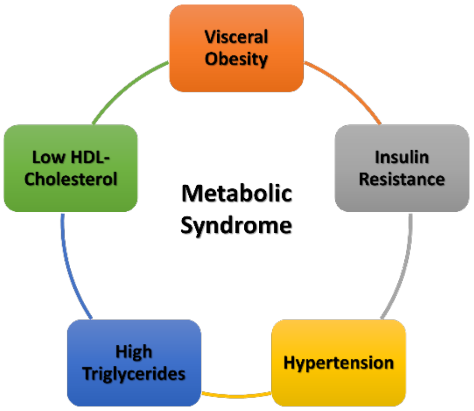

	Men	Women
Central obesity	Waist > 40 inches	Waist > 35 inches
Fasting triglycerides	> 150 mg/dL	> 150 mg/dL
Low HDL-C	< 40 mg/dL	< 50 mg/dL
Blood pressure	> 130/85	> 130/85
Fasting glucose	> 110 mg/dL	> 110 mg/dL

圖. 代謝症候群五大指標

腸道菌與代謝症候群的關係

腸道菌相對代謝疾病的影響：1)腸道菌分解膳食纖維(dietary fiber)與抗性澱粉(resistant starch)產生的短鏈脂肪酸(Short-chain fatty acids, SCFA)；2)革蘭氏陰性菌細胞膜上的脂多醣(Lipopolysaccharides, LPS)。在大量攝取高脂肪的食物情況下，腸道內革蘭氏陰性菌會大量增加，並產生許多的脂多醣。脂多醣是構成該菌外膜的主成分，除了完整細菌結構外並提供保護，與 CD14/Toll 樣受體 4 (TLR4)結合後會促進發炎反應，分泌多種細胞激素，造成發炎現象、脂肪生成增加與胰島素靈敏度下降，進而誘發代謝疾病產生。反之，如果大量攝取高纖食物，會讓分解纖維的好菌增生，產生大量的短鏈脂肪酸並發揮增強免疫等生理功能[4]。

高尿酸血症

尿酸(Uric acid)為普林代謝(Purine metabolism)的最終氧化產物，經由腎臟隨尿液排出體外。高尿酸血症患者的病症可能因尿酸結晶而引發痛風(gout)，導致四肢關節嚴重疼痛外，還可能引起腎臟疾病、泌尿系結石、心血管疾病、腦血管疾病等。因此，高尿酸血症也被認為是動脈硬化的危險因素之一。根據 2013 年的統計分析，台灣有 580 萬名患有高尿酸血症或痛風的患者。一般而言，女性血清尿酸的正常範圍上限為 360 μmol/L (6 mg/dL)，男性則為 400 μmol/L (6.8 mg/dL)。臨床研究指出同時具有高尿酸血症和糖尿病的患者，其血壓、血脂、膽固醇、BMI 值、肌酸酐和 HbA1c 水平顯著高於僅患有高尿酸血症的患者。顯示此病症會增加患者罹患其他慢性疾病的

風險。高尿酸血症是一種沒有早期症狀的代謝性疾病，多數患者在偶然進行全面身體檢查時才會意識到尿酸升高。

益生菌

乳酸菌(Lactic acid bacteria, LAB)又稱為益生菌(Probiotics)，一般來說被公認為安全且廣泛存在，使用或添加在許多食品、保健品中，益生菌(Probiotics)一詞起源於希臘語"for life"，對宿主健康有益的活微生物，益生菌及相關乳製品有悠久且安全的歷史，益生菌菌株的特異性與其功效有關，但並非每株菌株皆有相同功效，常見功效為:改善乳糖不耐症、預防或減緩幽門螺旋桿菌感染，減緩腸道障礙及調節免疫等。

腸道菌相檢測技術現況

次世代定序技術 (Next-Generation Sequencing, NGS)，同時擁有高通量以及高精確度的特性，能夠精確地的呈現高度複雜的微生物菌相圖譜[2]。以總基因體學(Metagenomics)搭配次世代定序，利用細菌的 16S 或真菌的 18S rDNA 來鑑定菌群種類與數量，是目前效率最高、最廣泛運用之菌相研究工具[3]。次世代定序技術 (NGS)針對糞便檢體做分析，能同時間高通量、極大量與最深度細微的高靈敏度，檢測了解人體腸道內最完整、最深度的腸道菌相改善狀況。

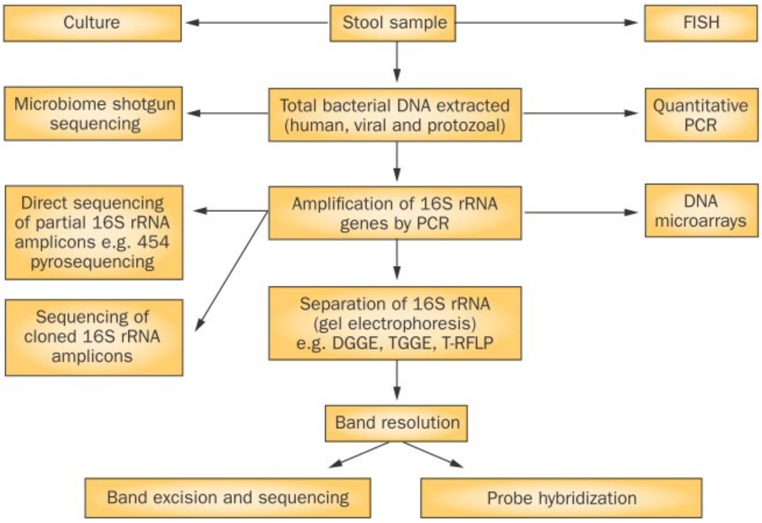

圖. 應用於菌相檢測的多項技術

目的

由於台灣的地理環境是一個海島，國人的飲食習慣多偏重海鮮及內臟類，加上熱情好客的風土民情，應酬飲酒頻繁，這些因素導致台灣的高尿酸血症盛行率名列前茅，長期下來就會造成腸道菌叢失衡，進而影響身體的代謝機能，本研究臨床主要觀察肝功能指數異常及高尿酸的受試者補充益生菌兩個月後，血液生化分析中肝腎功能指標及尿酸含量是否有改善，以及其他影響。

<u>**材料與方法:**</u>

<u>**試驗設計**</u>

根據降尿酸及保肝的試驗目的，將招募 120 位受試者為高尿酸同時具有肝指數過高之人群，受試者的基礎肝指數(GOT>38 IU/L，GPT>44 IU/L)，基礎尿酸數值，男性> 7mg/dL、女性> 6mg/dL。護肝、降尿酸系列(PRONULIFE® UriManage/ 迅酸清®)產品中所含有的菌株，分別為 *L. fermentum* TSF331、*L. reuteri* TSR332、*L. plantarum* TSP05。受試者分別於益生菌補充後第 0 天及第 60 天，進行身體基本測量(身高、體重、體脂肪、內臟脂肪、問卷)、血液生化數值(檢驗項目參考下圖)及糞便收集。

樣品種類	功能評估	檢驗項目
血清	肝臟	白蛋白 (ALB)
		總膽紅素 (T-Bil)
		麩胺酸轉移酶 (GGT)
		天門冬氨酸轉胺酶 (GOT)
		丙氨酸轉胺酶 (GPT)
		鹼性磷酸酶 (ALK-P)
	腎臟	肌酸酐 (creatinine)
		尿素氮 (BUN)
		尿酸
		血糖
	血脂	三酸甘油脂 (TG)
		總膽固醇 (T-CHOL)
		高密度脂蛋白膽固醇 (HDL-C)
		低密度脂蛋白膽固醇 (LDL-C)
	心血管疾病指數	乳酸脫氫酶 (LDH)
		肌酸磷化酶 (CPK)
其他	抗氧化指數	超氧化物歧化酶 (SOD)
		過氧化氫酶 (catalase)
		穀胱甘過氧化酶 (GPX)
	身體發炎指數	C反應性蛋白 (CRP)
		血清素 (serotonin)
		內毒素 (LPS)
		內毒素結合蛋白 (LBP)
		尿毒素<br>p-Cresyl Sulfate 和Indoxyl Sulfate

圖. 血液生化數值分析項目

<u>**生化數值檢驗**</u>

招募受試者並簽署受試者同意書，採集服用益生菌前的血清，並給予益生菌，請受試者依照試驗規劃食用 2 個月後，再採集服用後的血清，兩相對照生化數值檢驗，包括 glucose AC, GOT, GPT, BUN, Creatinine, TG, total cholesterol, HDL 與 LDL 等。

<u>**次世代定序腸道菌相檢驗**</u>

同樣採集服用益生菌前後的糞便檢體，依照「Preparing 16S Ribosomal RNA Gene Amplicons for the Illumina MiSeq System」標準操作程序，放大 16S rRNA 的 V3 及 V4 基因片段，用 Illumina MiSeq 次世代定序進行分析。

<u>**生物資訊分析**</u>

NGS 所得序列用 Illumina BaseSpace，以 Greengene 13.7 為資料庫，完整分析腸道內細菌的種類與數量，再利用 CLC Genomic workbench (Qiagen) 軟體分析，產生 OTU table、分析多樣性(alpha diversity , beta diversity)。

<u>**統計分析**</u>

利用 Prism 8 統計軟體進行統計分析，比較服用益生菌前後。並利用 R 語言 (R language) 以及 SPSS v25.0 統計軟體進行統計分析，當 $p < 0.05$ 認定具有統計意義。

参考文献

1. Thaiss CA, Zmora N, Levy M, Elinav E: The microbiome and innate immunity. Nature 2016, 535(7610):65-74.
2. Caporaso JG, Lauber CL, Walters WA, Berg-Lyons D, Huntley J, Fierer N, Owens SM, Betley J, Fraser L, Bauer M et al: Ultra-high-throughput microbial community analysis on the Illumina HiSeq and MiSeq platforms. ISME J 2012, 6(8):1621-1624.
3. Escobar-Zepeda A, Vera-Ponce de Leon A, Sanchez-Flores A: The Road to Metagenomics: From Microbiology to DNA Sequencing Technologies and Bioinformatics. Front Genet 2015, 6:348.
4. P.D. Cani, N.M. Delzenne, The role of the gut microbiota in energy metabolism and metabolic disease, Current pharmaceutical design, 15 (2009) 1546-1558.
