# Supplementary material for: Probiotic Lactobacillus fermentum TSF331, Lactobacillus reuteri TSR332, and Lactobacillus plantarum TSP05 improved liver function and uric acid management-A pilot study
Source: PLoS One. 2024 Jul 24;19(7):e0307181. doi: 10.1371/journal.pone.0307181 (PMC11268587; doi:10.1371/journal.pone.0307181)
Supplement: S2 Protocol — (PDF) [file pone.0307181.s003.pdf]

Trial study protocol

Abstracts

The impact of gut microbiota on human health has received increasing attention and expectations from the world. Probiotics are one of the most recognized and positive health materials for consumers. *Lactobacillus*/probiotics, the main raw materials of health foods most frequently purchased by domestic consumers in recent years, have always ranked the first. They have certain improvement effects in terms of gastrointestinal improvement, immune regulation, and blood lipid regulation. With the progress of human culture and lifestyle, many diseases of modern civilization have emerged one after another. Most of these diseases are caused by changes of lifestyle or diet, leading to the human body's metabolic imbalances, such as high blood sugar, high cholesterol, hypertension, hyperuricemia, liver dysfunction, and obesity. Gut dysbiosis and diseases associated with aging usually have such phenomenon like increased pathogenic bacteria, decreased probiotics decrease and declined gut microbiota diversity. The gut dysbiosis contributes to the aging gut and aging-related diseases in at least three aspects, including production of Lipopolysaccharides (LPS), reduced levels of short chain fatty acids (SCFAs), and inflamm-aging. Next-generation sequencing (NGS) technology has been employed to assess alterations in the composition of the human gut microbiota. The objective of this research is to investigate the impact of metabolism-regulating probiotics (*Lactobacillus fermentum* TSF331、*L. reuteri* TSR332、*L. plantarum* TSP05) on human biochemical profiles and intestinal flora through clinical trials conducted at the Aging and Disease Prevention Research Center (ADPRC) of Fooyin University. This project aims to identify disparities and enhancements in various facets of clinical validation, encompassing blood biochemical parameters and NGS analysis of intestinal microbiota, before and after probiotic supplementation.

Key word: Gut microbiota, Probiotics, Metabolic imbalances, SCFAs

Introduction

Metabolic syndrome

Metabolic syndrome refers to a cluster of risk factors that predispose individuals to cardiovascular diseases. These risk factors primarily encompass hypertension, dyslipidemia, diabetes, obesity, as well as abnormalities in uric acid levels and coagulation factors. The Third Report of the National Cholesterol Education Program Expert Panel (NCEP ATP III) in the United States defines metabolic syndrome as the presence of three or more of the following criteria in an individual.

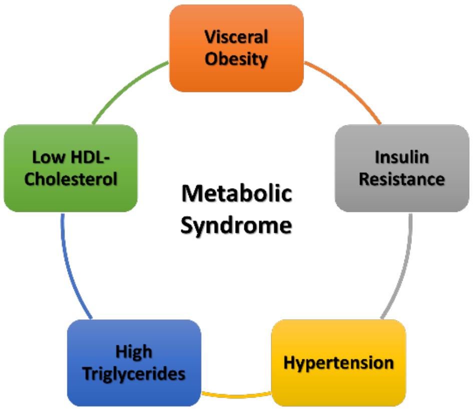

	Men	Women
**Central obesity**	Waist > 40 inches	Waist > 35 inches
**Fasting triglycerides**	> 150 mg/dL	> 150 mg/dL
**Low HDL-C**	< 40 mg/dL	< 50 mg/dL
**Blood pressure**	> 130/85	> 130/85
**Fasting glucose**	> 110 mg/dL	> 110 mg/dL

Figure: Five major indicators of metabolic syndrome

The Relationship Between Gut Microbiota and Metabolic Syndrome

Influence of gut microbiota on metabolic disorders includes: 1) gut microbiota-mediated fermentation of dietary fiber and resistant starch leading to the production of short-chain fatty acids (SCFAs), and 2) the presence of lipopolysaccharides (LPS) on the cell membrane of Gram-negative bacteria. In situations of excessive consumption of high-fat foods, there is a substantial proliferation of Gram-negative bacteria in the intestinal tract, leading to the abundant production of lipopolysaccharides. LPS constitute the primary component of the bacterial outer membrane, serving not only to maintain the integrity of the bacterial structure but also providing a protective function. When binding with CD14/Toll-like Receptor 4 (TLR4), LPS activates inflammatory responses, leading to the secretion of various cytokines. This cascade of events results in inflammatory manifestations, increased lipogenesis, and decreased insulin sensitivity, thereby inducing the onset of metabolic disorders. Conversely, substantial intake of high-fiber foods stimulates the proliferation of beneficial bacteria involved in fiber degradation. This leads to the production of abundant short-chain fatty acids, thereby exerting enhanced physiological functions, such as immune modulation [4].

Hyperuricemia

Uric acid is the final oxidized product of purine metabolism, excreted from the body through the kidneys in the urine. Individuals with hyperuricemia may experience symptoms that can precipitate gout, characterized by severe pain in the extremity joints due to the formation of uric acid crystals. Additionally, this condition may give rise to complications such as renal disorders, urolithiasis, cardiovascular diseases, and cerebrovascular disorders. Therefore, hyperuricemia is also considered one of the risk factors for arterial sclerosis. According to the statistical analysis in 2013, there are 5.8 million patients suffering from hyperuricemia or gout in Taiwan. In general, the upper end of the normal range of serum uric acid is 360 µmol/L (6 mg/dL) for women and 400 µmol/L (6.8 mg/dL) for men. In patients with hyperuricemia and diabetes at the same time, clinical studies have shown significantly higher levels of blood pressure, blood lipids, cholesterols, BMI value, creatinine, and HbA1c than in patients with hyperuricemia only. In other words, hyperuricemia increases the risk of getting other chronic diseases. Hyperuricemia is a metabolic disorder without early symptoms, and most of patients are not aware of the elevation of their uric acid until full physical examinations are done by chance.

Probiotics

Lactic acid bacteria (LAB), commonly known as probiotics, are generally acknowledged as safe and widely prevalent. They are utilized or incorporated into numerous food and dietary supplement formulations. The word 'Probiotics' originates from the Greek words 'for life,' referring to beneficial living microorganisms that contribute to the well-being of the host. Probiotics, along with related dairy products, have a long history of being considered safe. The efficacy of these strains is closely tied to their strain specificity; however, not every strain exhibits identical benefits. The common beneficial effects of probiotics include alleviating lactose intolerance, preventing or attenuating *Helicobacter pylori* infections, mitigating intestinal disorders, and modulating immune responses.

Current state of intestinal microbiota analysis techniques

Next-Generation Sequencing (NGS), characterized by both high throughput and high precision, facilitates the accurate representation of highly complex microbial community profiles [2]. The integration of metagenomics with NGS, utilizing bacterial 16S or fungal 18S rDNA, stands as the most efficient and widely employed approach for taxonomic identification and quantification of microbial communities [3]. NGS is employed for the analysis of fecal samples, enabling high-throughput, extensive, and in-depth analysis. This technique offers high sensitivity in detecting and comprehending the most comprehensive and profound alterations in the human intestinal microbiota.

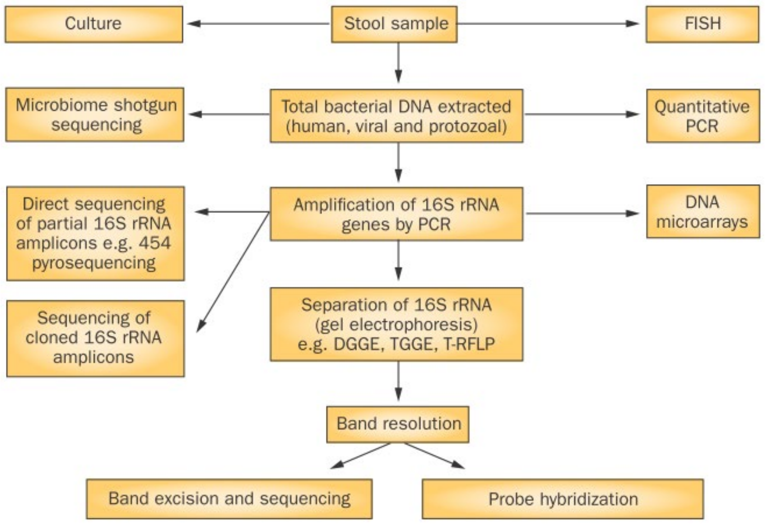

Figure: Multiple techniques applied in microbial community analysis

Objective

Due to Taiwan's geographical environment as an island, the dietary habits of the population tend to emphasize the consumption of seafood and organs. The prevalence of hyperuricemia in Taiwan is high, partly attributed to convivial cultural practices characterized by warm hospitality and frequent social drinking. Over the long term, this can lead to dysbiosis in the gut microbiota, subsequently impacting the body's metabolic functions. This clinical study primarily observes the impact of probiotic supplementation on subjects exhibiting abnormal liver function indices and elevated uric acid levels. After a supplementation period of two months, the study evaluates whether there are improvements in liver and kidney function parameters, as well as uric acid levels, through blood biochemical analysis. Additionally, the study explores other potential influences on these outcomes.

Materials and Methods

Study design

For the purpose of a trial focused on reducing uric acid levels and promoting liver health, we aim to recruit 120 participants who simultaneously exhibit elevated uric acid and elevated liver indices. Eligible participants will have baseline liver indices (GOT > 38 IU/L, GPT > 44 IU/L) and baseline uric acid values (male > 7 mg/dL, female > 6 mg/dL). The strains contained in the hepatoprotective and uric acid-lowering series (PRONULIFE® UriManage/ Quick Acid Clear®) products are *Lactobacillus fermentum* TSF331, *Lactobacillus reuteri* TSR332, and *Lactobacillus plantarum* TSP05, respectively. On days 0 and 60 following probiotic supplementation, subjects will undergo a comprehensive assessment, including anthropometric measurements (height, weight, body fat, visceral

fat, and questionnaire-based evaluation), blood biochemical parameters (as detailed in the accompanying figure), and fecal sample collection.

樣品種類	功能評估	檢驗項目
血清	肝臟	白蛋白（ALB）
		總膽紅素（T-Bil）
		麩胺酸轉移酶（GGT）
		天門冬氨酸轉胺酶（GOT）
		丙氨酸轉胺酶（GPT）
		鹼性磷酸酶（ALK-P）
	腎臟	肌酸酐（creatinine）
		尿素氮（BUN）
		尿酸
		血糖
	血脂	三酸甘油脂（TG）
		總膽固醇（T-CHOL）
		高密度脂蛋白膽固醇（HDL-C）
		低密度脂蛋白膽固醇（LDL-C）
	心血管疾病指數	乳酸脫氫酶（LDH）
		肌酸磷化酶（CPK）
其他	抗氧化指數	超氧化物歧化酶（SOD）
		過氧化氫酶（catalase）
		穀胱甘過氧化酶（GPX）
	身體發炎指數	C反應性蛋白（CRP）
		血清素（serotonin）
		內毒素（LPS）
		內毒素結合蛋白（LBP）
		尿毒素<br>p-Cresyl Sulfate 和Indoxyl Sulfate

Figure. Analysis parameters of blood biochemical values

Hematological biochemical parameters examination

Participants will be recruited and will sign informed consent forms upon agreement. Pre-probiotic serum samples will be collected before the ingestion of probiotics. Participants will be instructed to consume the probiotics according to the trial plan for a duration of two months. Following this period, post-probiotic serum samples will be collected. A comparative biochemical analysis will be conducted, encompassing parameters such as glucose AC, GOT, GPT, BUN, Creatinine, TG, total cholesterol, HDL, and LDL.

Next-generation sequencing (NGS) for gut microbiota profiling

The fecal samples will be collected before and after probiotic consumption, following the standard operating procedure for 'Preparing 16S Ribosomal RNA Gene Amplicons for the Illumina MiSeq System.' The amplification of the V3 and V4 gene segments of 16S rRNA is conducted, and subsequent analysis is performed using Illumina MiSeq next-generation sequencing.

Bioinformatics analysis

Analysis of intestinal bacterial diversity and abundance will be derived from NGS sequences using Illumina BaseSpace with Greengenes 13.7 as the reference database. Subsequent processing involves the utilization of CLC Genomic Workbench (Qiagen) software to generate an operational taxonomic unit (OTU) table and conduct alpha diversity and beta diversity analyses.

Statistical analysis

Prism 8 statistical software will be utilized for the comparative analysis of pre- and post-probiotic supplementation effects. Additionally, statistical analyses will be conducted using the R programming language and SPSS v25.0 software, with significance determined at $p < 0.05$.

References

1. Thaiss CA, Zmora N, Levy M, Elinav E: The microbiome and innate immunity. Nature 2016, 535(7610):65-74.
2. Caporaso JG, Lauber CL, Walters WA, Berg-Lyons D, Huntley J, Fierer N, Owens SM, Betley J, Fraser L, Bauer M et al: Ultra-high-throughput microbial community analysis on the Illumina HiSeq and MiSeq platforms. ISME J 2012, 6(8):1621-1624.
3. Escobar-Zepeda A, Vera-Ponce de Leon A, Sanchez-Flores A: The Road to Metagenomics: From Microbiology to DNA Sequencing Technologies and Bioinformatics. Front Genet 2015, 6:348.
4. P.D. Cani, N.M. Delzenne, The role of the gut microbiota in energy metabolism and metabolic disease, Current pharmaceutical design, 15 (2009) 1546-1558.
