# Supplementary material for: Probiotic Lactobacillus fermentum TSF331, Lactobacillus reuteri TSR332, and Lactobacillus plantarum TSP05 improved liver function and uric acid management-A pilot study
Source: PLoS One. 2024 Jul 24;19(7):e0307181. doi: 10.1371/journal.pone.0307181 (PMC11268587; doi:10.1371/journal.pone.0307181)
Supplement: S1 Table — (DOCX) [file pone.0307181.s004.docx]

**S1 Table. The blood biochemical profile on day 0.**

|  | **Placebo**  **(N=11)** | **TSF331**  **(N=14)** | **TSR332**  **(N=14)** | **TSP05**  **(N=13)** | **3 Mix**  **(N=15)** | **3 Mix**  **+PE0401 (N=15)** | ***P*-Value** |
| --- | --- | --- | --- | --- | --- | --- | --- |
| **Gender**  **(M/F)** | 6/5 | 8/6 | 8/6 | 8/5 | 8/7 | 8/7 | - |
| **Age**  **(year)** | 40.18  ± 10.30 | 38.21  ± 8.92 | 44.64  ± 14.40 | 44.92  ± 14.30 | 37.07  ± 5.78 | 37.60  ± 5.10 | 0.161 |
| **TBIL**  **(mg/dL)** | 0.78  ± 0.21 | 0.65  ± 0.26 | 0.87  ± 0.37 | 0.83  ± 0.39 | 0.65  ± 0.26 | 0.89  ± 0.42 | 0.198 |
| **GGT**  **(U/L)** | 23.36  ± 12.67 | 33.57  ± 19.00 | 32.00  ± 15.93 | 30.77  ± 5.05 | 26.53  ± 7.85 | 24.13  ± 21.89 | 0.382 |
| **ALKP**  **(U/L)** | 55.64  ± 10.81 | 69.50  ± 22.45 | 61.29  ± 24.84 | 64.54  ± 24.82 | 56.07  ± 16.66 | 55.20  ± 19.92 | 0.374 |
| **ALB**  **(g/dL)** | 4.53  ± 0.26 | 4.73  ± 0.51 | 4.56  ± 0.41 | 4.52  ± 0.46 | 4.50  ± 0.27 | 4.50  ± 0.27 | 0.676 |
| **CREA**  **(mg/dL)** | 0.86  ± 0.12 | 0.86  ± 0.13 | 0.77  ± 0.14 | 0.81  ± 0.15 | 0.81  ± 0.12 | 0.81  ± 0.12 | 0.545 |
| **BUN**  **(mg/dL)** | 13.62  ± 4.39 | 14.99  ± 5.20 | 14.36  ± 4.53 | 16.63  ± 5.19 | 13.67  ± 3.49 | 13.67  ± 3.49 | 0.384 |
| **GLU**  **(mg/dL)** | 85.82  ± 5.40 | 86.00  ± 9.17 | 86.21  ± 8.87 | 87.77  ± 8.13 | 84.87  ± 8.68 | 88.13  ± 10.35 | 0.916 |
| **TG**  **(mg/dL)** | 121.27  ± 15.13 | 126.29  ± 16.95 | 127.64  ± 19.76 | 132.23  ± 14.73 | 131.13  ± 15.40 | 128.67  ± 15.79 | 0.635 |
| **CHOL**  **(mg/dL)** | 185.18  ± 26.01 | 172.86  ± 38.95 | 182.50  ± 23.96 | 171.85  ± 30.96 | 169.73  ± 24.25 | 175.87  ± 28.76 | 0.719 |
| **LDL**  **(mg/dL)** | 115.67  ± 31.44 | 105.56  ± 30.84 | 93.26  ± 38.58 | 110.65  ± 47.04 | 94.46  ± 30.87 | 101.63  ± 36.24 | 0.574 |
| **HDL**  **(mg/dL)** | 51.87  ± 10.88 | 54.59  ± 10.44 | 54.67  ± 10.89 | 50.76  ± 11.12 | 48.21  ± 8.00 | 50.29  ± 10.01 | 0.491 |
| **Hs-CRP**  **(mg/dL)** | 0.23  ± 0.07 | 0.26  ± 0.17 | 0.34  ± 0.17 | 0.30  ± 0.18 | 0.18  ± 0.24 | 0.21  ± 0.19 | 0.185 |
| **LDH**  **(U/L)** | 107.27  ± 29.47 | 123.86  ± 30.94 | 102.21  ± 26.83 | 114.54  ± 26.92 | 106.20  ± 37.26 | 105.73  ± 28.71 | 0.455 |
| **CK**  **(U/L)** | 111.82  ± 58.58 | 119.36  ± 57.30 | 108.57  ± 50.09 | 85.38  ± 39.95 | 87.80  ± 19.37 | 102.93  ± 59.61 | 0.404 |

Data are presented as mean ± SD of the results from each subject. The *P*-value presented the difference among groups using One-way ANOVA.
